# Phytochemical and pharmacological review of *Lagenaria sicereria*

**DOI:** 10.4103/0975-9476.74431

**Published:** 2010

**Authors:** Rakesh P. Prajapati, Manisha Kalariya, Sachin K. Parmar, Navin R. Sheth

**Affiliations:** *Saurashtra University, Rajkot, Gujarat, India*

**Keywords:** Chemical constituents, cucurbitaceae, ethnomedical uses, Lagenaria *siceraria*, pharmacological profile

## Abstract

*Lagenaria siceraria* (Molina) standley (LS) (Family: Cucurbitaceae) is an annual herbaceous climbing plant with a long history of traditional medicinal uses in many countries, especially in tropical and subtropical regions. Since ancient times the climber has been known for its curative properties, and has been utilized for treatment of various ailments, including jaundice, diabetes, ulcer, piles, colitis, insanity, hypertension, congestive cardiac failure (CCF), and skin diseases. Its fruit pulp is used both as an emetic and purgative, and for its cooling, diuretic, antibilious, and pectoral properties. Boiled in oil this pulp is used to treat rheumatism and insomnia. A wide range of chemical compounds including sterols, terpenoids, flavonoids, and saponins have been isolated from the species. Its extracts have been found to possess various pharmacological activities. Below, we give a comprehensive review of its ethnomedical uses, chemical constituents, and pharmacological profile as a medicinal plant. Particular attention is given to its analgesic, anti-inflammatory, antihyperlipidemic, diuretic, hepatoprotective, anthelmintic, and antibacterial effects so that its potential uses in pharmaceutics can be better evaluated.

## INTRODUCTION

Traditional systems of medicines have always played important roles in meeting global healthcare needs. They continue to do so today and will also play major roles in the future. Systems of medicine considered Indian in origin, or systems of medicine which have come to India from abroad and been assimilated into Indian culture are collectively known as Indian Systems of Medicine (ISM). India has the unique distinction of having six recognized systems of medicine in this category: Ayurveda, Yoga and Naturopathy, Unani, Siddha, and Homeopathy.[1]

Among them, Ayurveda has been practiced for thousands of years. Considerable research on the pharmacognosy, chemistry, pharmacology, and clinical therapeutics of Ayurvedic medicinal plants has been carried out. Natural products, including those from plants, animals, and minerals have been the basis of its treatment of disease. The currently dominant system, modern medicine or ‘allopathy’ has gradually developed and over the years come to be accepted through scientific research and observation. However, the ultimate basis for its development lies in traditional medicine and therapies.[2]

In explaining medicinal plants, *Rigveda* dates them back three yugas prior to the existence of animal life on earth. This indicates the importance it attributed to medicinal plants. Ayurvedic texts, from the *Samhitas* to the *Nighantus*, identify about 2000 species of plants and explain their properties. But India’s 4,635 ethnic communities include one million folk healers using an estimated 8000 or more species of medicinal plants. Their rural households have little or no financial means to buy drugs off the shelf for medical care. Such folk medicines are the first response to simple ailments. Their economic and therapeutic potential makes standardization, documentation, and conservation of medicinal plants of vital importance

Selecting the right scientific and systematic approach to biological evaluation of plant products, based on their use in traditional medicine is the key to ideal development of new drugs from plants. One such plant is Lagenaria siceraria (Molina) standley (LS) (Family: Cucurbitaceae). It is a large, softly pubescent, annular, climbing or trailing herb growing throughout the India.[3]

The entire plant is recognized to be beneficial in ethnic systems of medicine. The fruit is sweet, diuretic, antipyretic, antibilious, tonic for the liver, vulnerary, and antiperiodic. It can cure blood diseases in persons of pitta constitution; muscular pain and dry cough. In Punjab, the pulp is applied to the soles of the feet of those with “burning feet.” The seeds are fattening, cooling, anthelmintic, and a brain tonic; they can cure cough, fever, scalding urine, and earache; they also reduce inflammation (Unani). Their oil can be applied for headache. The rind of the fruit is good for piles, while its ash is styptic and vulnerary. The root is applied in the treatment of dropsy.[4]

## PLANT PROFILE

LS is also known as *Lagenaria leucantha* Rusby and *Lagenaria vulgaris* Seringe. Its common names include bottle gourd (Eng.); alabu (Sanskrit); lauki or ghia (Hindi); dudhi or tumbadi (Gujarati); sorakkai (Tamil); chorakkaurdu (Malayalam); and ghiya (Urdu). Geographically it occurs throughout India and is now cultivated worldwide. It is generally accepted that LS was indigenous to Africa and that it reached temperate and tropical areas in Asia and the Americas about 10,000 years ago.[5]

## PROPERTIES AND ACTIONS MENTIONED IN AYURVEDA[5]

*Rasa* : *Madhura (sweet)*

*Guna* : *Snigdha* (viscous)

*Virya* : *sita* (cool)

*Vipaka* : *Madhura* (pleasant)

*Karma* : *Pittahara, Bhedaka, Hrdya, Vrsya*

## BOTANICAL DESCRIPTION

### Parts used[3,5,6]

All parts of the plant including ripe fruits, leaves, stem, and flowers may be used for the treatment of various ailments as described above.

### Morphology

**Figure 1:**
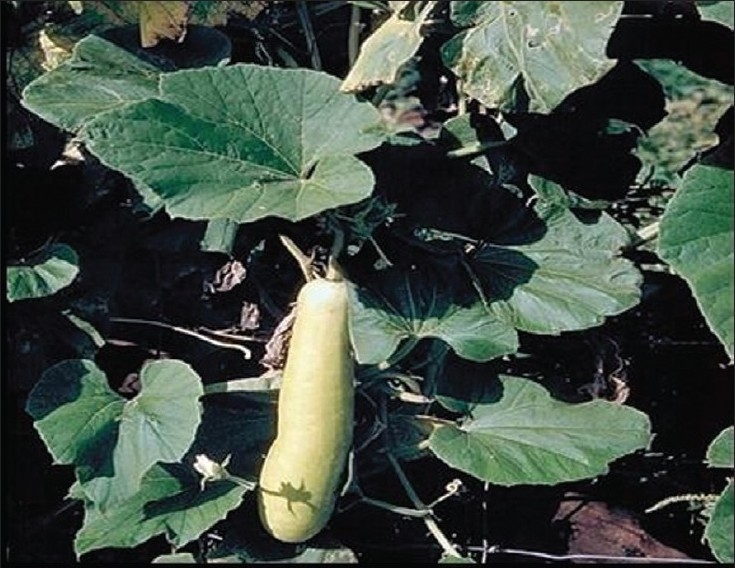
*Lagenaia siceraria* plant

Vigorous annual herb. *Stems* are prostrate or climbing, angular, ribbed, thick, brittle, softly hairy, upto 5 m long, cut stems exude no sap. *Leaves* are simple, up to 400 mm long and 400 mm broad, long petioled, 5-lobed, cordate, pubescent, shortly and softly hairy, broadly egg-, kidney-, or heart-shaped in outline, undivided, angular, or faintly 3–7 lobed, lobes rounded, margins shallowly toothed, crushed leaves nonaromatic. Leaf stalks up to 300 mm long, thick, often hollow, densely hairy, with two small, lateral glands inserted at the leaf base. Tendrils split in two. Flowers are stalked (female flower stalks shorter than male), solitary, unisexual, axillary, monoecious; petals 5, crisped, cream or white colored with darker veins, pale yellow at the base, obovate, up to 45 mm long, opening in the evenings, soon wilting. Fruits are large, variable, cylindrical, flask-shaped or globose with a constriction above the middle; fleshy, densely hairy, indehiscent, green, maturing yellowish or pale brown, pulp drying out on ripening, leaving a thick, hard, hollow. Seeds are many, embedded in a spongy pulp, compressed, with two flat facial ridges, in some variants rather irregular and rugose [Figure 1].[7]

### Traditional uses

The fruits, leaves, oil, and seeds are edible and used by local people as folk medicines in the treatment of jaundice, diabetes, ulcer, piles, colitis, insanity, hypertension, congestive cardiac failure, and skin diseases. The fruit pulp is used as an emetic, sedative, purgative, cooling, diuretic, antibilious, and pectoral. The flowers are an antidote to poison. The stem bark and rind of the fruit are diuretic. The seed is vermifuge. Extracts of the plant have shown antibiotic activity. Leaf juice is widely used for baldness.[4,7,8]

In Curacao, a leaf decoction is taken for flatulence. Decoctions containing a combination of *Langenaria siceraria* and *Rivina humilis* are given for gas in pregnancy. In combination with garlic, a decoction is taken for gas pain in the heart area. Leaves with salt or coconut oil are often used as poultices for mange, skin irritation, and tumors. A poultice of the crushed leaves has been applied to the head to treat headaches. Taken with *Achyranthes* spp., the seed is used to treat aching teeth and gums, boils, etc. Pulverized seed kernels are taken to expel intestinal worms. In many parts of China, 3 g per day has been used as a single treatment for diabetes mellitus.[3,4,7,8]

### Home remedies of LS (lauki) juice

LS juice is an excellent remedy for heart problems, digestive and urinary disorders, and in diabetes. Dietary fiber present in LS helps in constipation, flatulence, and even in piles. Topical application of a mixture of LS juice and sesame oil on scalp gives beneficial results in baldness (hair loss). The juice also shows better effects in the treatment of insomnia, epilepsy, and other nervous diseases. Moreover it helps break up calculus (stones) in the body. In summer or hot conditions, LS juice prevents excessive loss of sodium, satiating thirst, and giving a cooling effect.[7]

### Commercial application

Commercially the shell of well-ripened fruits is very hard and can be used for many purposes such as bottles, bowls, musical instruments, etc.[3]

### Phytochemistry

Fruits of the sweet variety contain carbohydrate (2.5%), protein (0.2%), fat (0.1%) (ether extract), fibers (0.6%), mineral matter (0.5%), calcium and phosphorous (<0.01%). Other mineral elements reported to be present include iron (0.7 mg/100 g), sodium (11.0 mg/100 g), and iodine (4.5 μg/kg). The fruit also contains 15.8 μg/g retinol. The amino acid composition of the fruit is as follows: leucines, 0.8; phenylalanine, 0.9; valine, 0.3; tyrosine, 0.4; alanine, 0.5; threonine, 0.2; glutamic acid, 0.3; serine, 0.6; aspartic acid, 1.9; cystine, 0.6; cystiene, 0.3; arginine, 0.4; and proline, 0.3 mg/g. The edible portion contains: thiamine, 44 μg; riboflavin, 23 μg; niacin 0.33 mg; and ascorbic acid, 13 mg/100 g. It contains of choline.[9] The fruit is considered a good source of vitamin C, β-carotene, vitamin B-complex, pectin, and also, at 16.02 mg/g (dry basis), to contain the highest level of choline - a lipotropic factor.[9,10] Fruits also contain two triterpenoids, 22-deoxocurcubitacin-d, and 22-deoxoisocurcubitacin d. The fruit skin contains crude protein, 17.5%; cellulose, 18.1%; and lignin, 8.0%.[7,9,10]

The seeds contain steroidal moieties like avenasterol, codisterol, elesterol, isofucasterol, stigmasterol, sitosterol, compesterol, spinasterol; and sugar moieties including rhamnose, fructose, glucose, sucrose, raffinose[9] and saponin.[7,10] Seed kernels are rich in iron, potassium, sulfur, and magnesium and particularly rich in copper (28.3 ppm); They can be used as a dietary supplement.[10,11]

### Flavonoids

**Figure 2:**
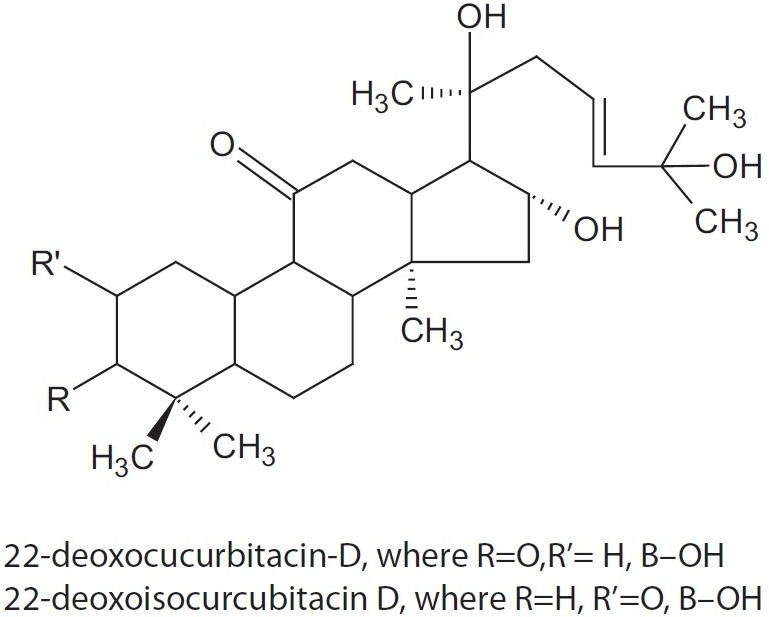
Structure of triterpenoids (22-deoxocurcubitacin-D and 22-deoxoisocurcubitacin-D) isolated form *L. siceraria*

Miroslawa and Cisowski (1995) isolated 4-C-glycosylflavone: 7-0-glucosyl-6-C-glucoside apigenin, 6-C-glucoside apigenin, 6-C-glucoside luteolin, and 7,4’-O-diglucosyl-6-C-glucoside apigenin from the plant and identified them by the spectroscopic analysis: UV, FD-MS, LSI-MS, H-NMR, C-NMR, melting point, and enzymatic hydrolysis. Moreover, using high-performance liquid chromatography, they also determined that the flavonoids in LS fruits are mainly isovitexin, isoorientin, saponarin, and saponarin 4’-O-glucoside[12][Figure 2, Table 1].

**Table 1:** Structural variation in flavone *C*-glycosides isolated from *L. siceraria*

Compound	R1	R2	R3	R4	R5
Vitexin	H	Glucose	H	H	H
Isovitexin	Glucose	H	H	H	H
Saponarin	Glucose	H	Glucose	H	H
Saponarin *C- O*- glucoside	Glucose	H	Glucose	H	Glucose
Saponarin Caffiec ester	Glucose	H	Caffioyl glucose	H	H
Isoorientin	Glucose	H	H	OH	H
Lutonarin	Glucose	H	Glucose	OH	H

### Protein

Wang *et al*. isolated lagenin, a novel ribosome-inactivating protein with ribonucleolytic activity, from seeds of the plant. In their study, the seeds of fruits were extracted with water and the extract was lyophilized. The lyophilized extract was chromatographed on a cellulose column in 10 mM Tris-HCl buffer (pH 7.2). The unadsorbed fraction was applied to an Affi-gel Blue gel column, previously equilibrated with the same buffer. After removal of unadsorbed materials, the adsorbed proteins were eluted with 1.5 M sodium chloride (NaCl) in the Tris-HCl buffer. After dialysis, the adsorbed fraction was loaded on CM-Sepharose CL-6B column which had been equilibrated with and was eluted with the same buffer. After elution of unadsorbed proteins, the column was eluted with a gradient of 0.1 M NaCl in 10 mM Tris-HCl buffer (pH 7.2). The fraction eluting at about 0.55 M NaCl, which represented pure ribosome inactivating protein (RIP), inhibited cell-free translation in a rabbit reticulocyte system with an IC_50_ of 0.21 nM and exerted ribonuclease activity on yeast t-RNA with an activity of 45 U/mg. The RIP was designated lagenin. It possessed a molecular weight of 20 kDa, smaller than the range of 26-32 kDa reported for other RIPs.[13]

### Triterpenes

**Figure 3:**
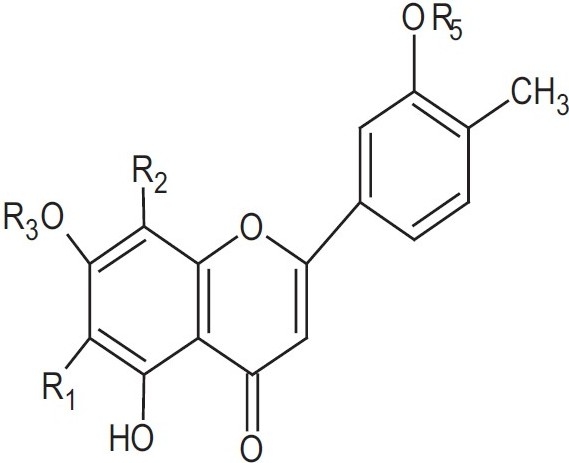
Structure of flavone *C*-glycosides from *L. siceraria*

**Figure 4:**
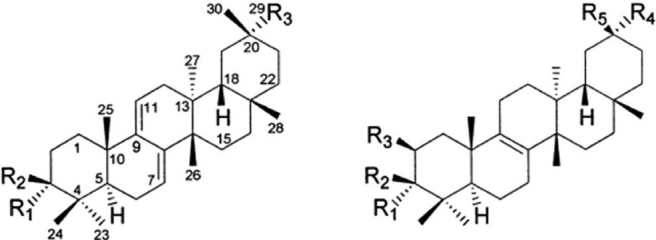
Structures of triterpenes isolated from *L. siceraria*

Chen *et al*, isolated four new d:*C*-friedooleanane-type triterpenes, 3 b-*O*-(*E*)-feruloyl-D:*C*-friedooleana-7, 9 (11)-dien-29-ol (1), 3b-*O*-(*E*)-coumaroyl-D:*C*-friedooleana-7,9(11)-dien-29-ol (2), 3b-*O*-(*E*) coumaroyl-d:*C*-friedooleana-7,9 (11)-dien-29-oic acid (3), and methyl 2 b,3 b-dihydroxy-D:C-friedoolean-8-en-29-oate (6), together with five known triterpenes with the same skeleton, 3-epikarounidiol (4), 3-oxo-d:C-friedoolena-7, 9 (11)-dien-29-oic acid (5), bryonolol (7), bryononic acid (8), and 20-epibryonolic acid (9) from the methanol extract of the stems of plant. Compounds 3 and 9 showed significant cytotoxic activity against the SK-Hep 1 cell line with IC_50_ values of 4.8[14] [Figure 3 and 4, Table 2].

**Table 2:** Structural variation in various triterpenes isolated from *L. siceraria*

Sr. No	R1	R2	R3	Sr. No.	R1	R2	R3	R4	R5
1	H	*O-(E)*-feruloyl	CH_2_OH	6	H	OH	OH	COOCH_3_	CH_3_
2	H	*O-(E)*-coumaroyl	CH_2_OH	7	H	OH	H	CH_2_OH	CH3
3	H	*O-(E)*-coumaroyl	COOH	8	---	=O	H	COOH	CH_3_
4	H	OH	CH_2_OH	9	H	OH	H	CH_3_	CH_3_
5	---	=O	COOH	10	H	OH	H	COOCH_3_	CH_3_

### Volatile essential oil

In 2009, Chatterjee isolated volatile principles by steam distillation and analyzed them by gas chromatography/mass spectrometry (GC/MS). Aliphatic aldehydes such as octanal, nonanal, and decanal with fruity, floral, and citrus odors dominated the volatile profile of bottle gourd. Many aliphatic aldehydes were identified in this oil such as octanal, nonanal, and decanal. These possess strong fruity, citrusy, and floral odors and low odor thresholds, and may play important roles in giving the fruit its characteristic aroma. Additionally, glycosidic precursors of bottle gourd including 1,4-benzenediol (12.51%); 2-pentadecyn-1-ol (17.87%); 9,12-octadecadienal; and fatty acids such as palmitic acid and stearic acid that dominated the volatile aroma profile may also contribute to the fatty odor of the vegetable. This constituted the first report of bound aroma precursors being present in the fruit.[15]

### Carbohydrates

Ghosh *et al*. isolated a water-soluble polysaccharide, methyl galacturonosyl-methoxyxylan from the stem of the plant. Structural studies found it to be constituted of equal proportions of methyl d-galactouronate, 2-O-methyl-d-xylose, and d-xylose. On the basis of total acid hydrolysis, methylation analysis, periodate oxidation, NMR studies, and matrix-assisted laser desorption/ionization (MALDI-MS) analysis, the structure of the repeating unit of the polysaccharide was determined to be.[16]

**Table:** 

A	B	C

4)-α-D-GalpA6Me-(1 3)-2-O-Me-β-d-Xylp-(1 2)-β-D-Xylp-(1

## ETHNOPHARMACOLOGY

### Bioactivity

The fruits are edible and traditionally used in the treatment of jaundice, diabetes, ulcer, piles, colitis, insanity, hypertension, cngestive cardiac failure, and skin diseases. It is used as a emetic, purgative, cooling, sedative, antibilious, and pectoral. Its pulp, boiled in oil is used to treat Rheumatism.

## PHARMACOLOGICAL PROPERTIES

### Analgesic and anti-inflammatory activity

Analgesic and anti-inflammatory effects of LS fruit juice extract were investigated in rats and mice. The juice extract was studied for its analgesic effect on acetic acid induced writhing and formalin pain tests in mice. Anti-inflammatory effects were investigated employing acute inflammatory models, i.e., ethyl phenylpropionate induced ear edema, carrageenan- and arachidonic acid induced hind paw edema, and also the albumin-induced paw edema in rats. Extract (150 -300 mg/kg, p.o.) exhibited a dose-dependent inhibition of writhing and also showed a significant inhibition of both phases of the formalin pain test, but with a less intense effect on the first phase than on the second. The extract’s effects were significantly lower than those produced by morphine (10 mg/kg) and aspirin (300 mg/kg) in the same tests. The extract elicited significant inhibitory effect on ear edema formation at 30 min, 1 h, and 2 h after EPP injection. In other acute inflammatory models, the extract significantly inhibited carrageenan- and arachidonic acid induced hind paw edema. LS juice also caused inhibition of albumin-induced paw edema over a period of 90 min.[17]

### Antihyperlipidemic activity

Antihyperlipidemic activity of the fruit extracts in triton-induced hyperlipidemic rats and hypolipidemic effect in normocholesteremic rats were investigated. In the study, four different extracts viz. petroleum ether, chloroform, alcoholic, and aqueous extracts were prepared. Oral administration of the extracts dose dependently inhibited the total cholesterol, triglycerides, low-density lipoproteins level, and significantly increased the high-density lipoproteins level. Both the chloroform and alcoholic extracts exhibited significant effects compared others. However, the petroleum ether extract did not show significant effects.[18]

### Diuretic activity

Diuretic activity of vacuum dried juice extract and methanol extract of the fruits was evaluated in albino rats. Different parameters viz. total urine volume, urine concentration of electrolytes such as sodium, potassium, and chloride were evaluated. Rats treated with vacuum dried juice extract and methanol extract (100 -200 mg/kg; p.o.) showed higher urine volume compared to respective controls. Both juice and methanol extract exhibited dose-dependent increase in the excretion of electrolytes when compared to controls. The elevated diuretic potentials of juice and methanol extract were statistically significant and comparable to that of the standard diuretic agent furosemide (20 mg/kg; i.p.).[19]

### Anthelmintic activity

Bottle gourd seeds showed *in vitro* anthelmintic activity against *Pheretima posthuma*. According to the author’s study, various concentrations (10 -100 mg/ml) of seed extracts were tested in the bioassay, which involved determination of time of paralysis and time of death of the worms. Piperazine citrate (10 mg/ml) was included as standard reference and distilled water as control. Of all the extracts, those using methanol and benzene showed significant paralysis, and also caused death of worms especially at the higher concentration of 100 mg/ml, compared to standard.[20]

### Antihepatotoxic activity

Antihepatotoxic activity of different fractions of the ethanolic extract of the fruit was determined by oral administration to different groups of rats using the CCl4-induced hepatotoxicity test. During the study, every fraction showed significant activity at dose of 250 mg/kg. Of all fractions, that in petroleum ether exhibited a comparatively higher activity. Furthermore two steroids isolated from the petroleum ether fraction were identified as fucosterol and campesterol.[21]

### Immunomodulatory activity

Immunomodulatory effects of n-butanol-soluble and ethyl acetate soluble fractions of successive methanolic extracts of the fruits were evaluated in rats. Oral administration of these fractions at doses 100 -500 mg/kg significantly inhibited delayed-type hypersensitivity reaction in rats. A dose-dependent increase in both primary and secondary antibody titer was observed. Fractions also significantly increased both white blood cell and lymphocyte count.[22]

### Antistress and adaptogenic property

Lakshmi and Sudhakar[23] evaluated the antistress potential of ethanolic extracts of fruits in albino Wistar rats and investigated the influence of forced swimming endurance stress on swimming endurance time, organ weights, and changes in biochemical parameters in rats. They investigated the acute heat stress induced changes in biochemical parameters, adrenal gland weight, and stress-induced perturbations in blood cell counts in rats. As a standard reference drug, *withania somnifera* was used. Pretreatment with the extract at different doses significantly (P <0.05) amelioratedstress-induced variations in the following biochemical parameters in these stress models: serum glucose, triglyceride, cholesterol and cortisol levels, blood cell counts, and organ weights. Extract-treated animals also showed increase in swimming endurance time. LS prolonged swimming time and ameliorated stress-induced changes in both stress models. This ability suggests both antistress and adaptogenic properties.[23]

### Antimicrobial activity

In Ethiopian traditional medicine, LS is widely used for treatment of skin disorders. Goji evaluated antimicrobial activity of methanolic extracts of the leaves, seeds, and fruit-flesh of L. siceraria (Cucurbitaceae) using the agar-well diffusion method. Results revealed LS extract to show activity against Pseudomonas aeruginosa and Streptococcus pyogenes, but not against clinical isolates of S. aureus and Escherichia coli. Thus LS can be used to treat various skin disorders.[24]

### Antioxidant activity

Deshpande *et al*, investigated free radical scavenging activity of the fruit. In their study, fruits were collected and the epicarp, mesocarp, and pulp containing seeds were separated. Each of them was extracted with different solvents in increasing order of polarity, using Soxhlet apparatus and serial extraction technique. All extracts were assessed by 1,1-dphenyl-2-picrylhydrazyl (DPPH) assay. Maximum antioxidant activity was observed for the acetone extract of the fruit epicarp. Chemical investigation revealed that free-radical scavenging activity may be due to ellagitannins present in the epicarp acetone extract.They also evaluated the effect of ethanolic extract of LS fruits against disorders, where free radicals play a major role in pathogenesis, and found it very effective as a hepatoprotective, antioxidant, antihyperglycemic, immunomodulatory, antihyperlipidemic, and cardiotonic agent.[25]

Dixit *et al*. studied antioxidant potential of fruit peel extract in carbon tetrachloride and hydrogen peroxide induced lipid peroxidation (LPO) in liver tissues in mice. In another experiment, an *in vivo* study was performed to evaluate three different concentrations of peel extract to select the most effective safe dose for regulation of hepatic LPO, thyroid function, and glucose metabolism. Out of 50, 100, and 200 mg/kg of peel extract, 100 mg/kg was found to be safest and most effective, as it could inhibit the levels of serum thyroxine, triiodothyronine, and glucose as well as hepatic LPO. Finally the antithyroidal, antiperoxidative, and glucose inhibitory potential of the peel extract were tested in T4-induced hyperthyroid animals. After 21 days of treatment, reductions in concentrations of serum thyroid hormones, glucose, and hepatic LPO were observed, with parallel increases in antioxidants such as superoxide dismutase, catalase, and glutathione, indicating the efficacy of the peel in hyperthyroidism, hyperglycemia, and hepatic LPO.[26]

## DISCUSSION AND CONCLUSIONS

L. *siceraria* is a well-known plant used in ISM; in addition, folk medicine also claims uses especially in cardiac and hepatic diseases, ulcer, etc. Recently LS fruit has become widely cultivated in India, Sri Lanka, China, and many other countries for its culinary and medicinal uses. It is also very important in a number of diseases for which there are considerable scientific reports and data. Chemically, LS contains various biologically active phytoconstituents including flavonoids, saponins, triterpenes, and volatile principles. It may thus be considered an important gift from Ayurveda to mankind.
